# Prediction of localization and interactions of apoptotic proteins

**DOI:** 10.1186/1423-0127-16-59

**Published:** 2009-07-06

**Authors:** Miroslav Vařecha, Michal Zimmermann, Jana Amrichová, Vladimír Ulman, Pavel Matula, Michal Kozubek

**Affiliations:** 1 Centre for Biomedical Image Analysis, Faculty of Informatics, Masaryk University, Botanická 68a, Brno 602 00, Czech Republic

## Abstract

During apoptosis several mitochondrial proteins are released. Some of them participate in caspase-independent nuclear DNA degradation, especially apoptosis-inducing factor (AIF) and endonuclease G (endoG). Another interesting protein, which was expected to act similarly as AIF due to the high sequence homology with AIF is AIF-homologous mitochondrion-associated inducer of death (AMID). We studied the structure, cellular localization, and interactions of several proteins *in silico *and also in cells using fluorescent microscopy. We found the AMID protein to be cytoplasmic, most probably incorporated into the cytoplasmic side of the lipid membranes. Bioinformatic predictions were conducted to analyze the interactions of the studied proteins with each other and with other possible partners. We conducted molecular modeling of proteins with unknown 3D structures. These models were then refined by MolProbity server and employed in molecular docking simulations of interactions. Our results show data acquired using a combination of modern *in silico *methods and image analysis to understand the localization, interactions and functions of proteins AMID, AIF, endonuclease G, and other apoptosis-related proteins.

## Background

During some forms of apoptosis the mitochondrial outer membrane becomes depolarized and partially permeable to proteins. This results in a massive nonspecific release of hydrophilic proteins from the intermembrane space into the cytoplasm [1]. Among these proteins are apoptosis-inducing factor (AIF) and endonuclease G (endoG). The release of these proteins results in activation of the apoptotic caspases, degradation of nuclear DNA, and cell death [2,3]. However, both AIF and endoG have been found to directly participate in DNA degradation in a caspase-independent way [4]. The protein AIF-homologous mitochondrion-associated inducer of death (AMID), which is probably not located in the mitochondrion, shares sequence homology with AIF and exerts similar apoptotic effects on nuclear chromatin [5]. Interestingly, endoG, AIF and AMID have all been found to influence chromatin changes during apoptosis [6].

EndoG is a mitochondrial nuclease with a molecular weight of 30 kDa. Its N-terminus contains a mitochondrial localization sequence (MLS), which is cleaved upon successful transport of the endoG precursor polypeptide across the outer mitochondrial membrane. EndoG migrates from mitochondria into the nucleus after apoptogenic stimuli [7,8]. Addition of endoG to isolated cell nuclei resulted in cleavage of the chromatin into large fragments (~50 kbp) and subsequently into inter- and intra-nucleosomal-size fragments with periodically repeated single-stranded breaks. The first phase of endoG activity equates with the large-scale degradation of DNA during apoptosis, but the second phase would not seem to be able to generate the characteristic "laddered" fragmentation of chromatin observed in apoptotic nuclei. This may suggest that endoG normally interacts with other nucleases. Indeed, cooperation between endoG, DNase I and exonuclease III has been shown to occur only on isolated dsDNA [6]. Another proposed interaction partner for endoG was found by protein analytic *in vitro *methods to be flap endonuclease 1 [9], but it was not yet shown in living or fixed cells as many other possible interactions mentioned here.

AIF (also known as AIFM1 or PDCD8) is an evolutionary conserved flavoprotein. It shares a high degree of sequence homology with bacterial, plant, and fungal oxidoreductases. The human AIF is expressed as a precursor polypeptide of molecular weight ~67 kDa. This precursor contains an N-terminal MLS, which is cleaved, and the active AIF (~57 kDa) is created in the mitochondrial intermembrane space [10]. AIF is probably bound by its N-terminus to the surface of the inner mitochondrial membrane [11,12]. The function of AIF in the mitochondrion under non-apoptotic conditions is not clear, but there is evidence that AIF may serve to sequester free radicals and that it can play important role in oxidative phosphorylation [13,14]. However, human AIF is also able to induce apoptosis [10]. None of these effects could be inhibited by the pan-caspase inhibitor z-VAD-fmk, thus they are caspase-independent [15]. Translocation of AIF into the nucleus occurs during apoptosis [15,16]. The *C. elegans *homologue of AIF, known as WAH-1, was shown to induce apoptosis and to migrate from mitochondria to the nucleus, where it interacts with a nuclease, CPS-6 (a homologue of the mammalian endoG), and together they mediate chromatin DNA degradation [17]. There is no clear evidence as to how mammalian AIF is involved in the process of chromatin degradation, but AIF can physically interact with DNA and RNA [18]. Interaction of human AIF and endoG, in analogy to what happens in *C. elegans*, has not yet been shown, although protein analysis *in vitro *results suggest its possibility [9]. However, other important proteins have been proposed to interact with AIF, namely cyclophilin A [19] and heat shock 70 kDa protein 1A (HSP70-1) [20]. Cyclophilin A is involved in transport of AIF into the nucleus and may be also involved in chromatin degradation [19,21]. HSP70-1 binds AIF in the cytoplasm and blocks its transport into the nucleus [22]. Another protein, DNA topoisomerase II α, was found to be involved in chromatin degradation during caspase-independent apoptosis and is therefore one of our candidate proteins that may interact with AIF in nucleus [23].

AMID (also known as AIFM2 or PRG3) is a flavoprotein with amino acid sequence similarity to NADH oxidoreductases in all species, as well as to AIF (22% identity). In contrast to AIF, AMID does not contain a recognizable MLS [5]. AMID was found to associate with the outer mitochondrial membrane [5] or to be freely distributed in the cytoplasm [24]. Apoptosis induced by AMID is regulated by p53, is caspase-independent, and is not affected by overexpression of the Bcl-2 protein [5]. Artificially induced overexpression of AMID caused chromatin condensation at the nuclear periphery and formation of apoptotic bodies [24]. However, this was not confirmed by another study [5]. Overexpression of AMID also caused a loss of structurally differentiated mitochondria [5]. However, recent results did not show that AMID acts similarly to AIF and it's localization was also questioned [25,26].

Therefore, in this work we use modern *in silico *methods for sequence analysis, predicting the subcellular localization of proteins, prediction of interactions, molecular modeling and docking and we combined these methods with fluorescence microscopic imaging of endoG, AIF, AMID, and other apoptotic proteins in living or fixed cells to analyze their localization and interactions.

## Materials and methods

### Cell culture

Human bone osteosarcoma U-2 OS cells were grown in minimal essential medium containing Earle's balanced salts, L-glutamine, non-essential amino acids, 1.5 g/l NaHCO_3_, 10% fetal bovine serum, 100 U/ml of penicillin and 100 μg/ml of streptomycin at 37°C in a 5% CO_2 _atmosphere.

### Plasmid DNA preparation and transfection

The plasmid constructs carrying the genes for endoG-EYFP, AIF-tHcRed, and AMID-tHcRed fluorescent fusion proteins were described previously [25]. Cells were transfected using FuGENE 6 (Roche). In cases of transient transfection, the transfected cells were used for experiments 24 hours after transfection. Stably transfected cell cultures were selected using 600 μg/ml of geneticine (G418) for 14 days.

### Prediction of protein's cellular localization

Prediction analysis was conducted using amino acid sequences of the endonuclease G protein precursor (GenBank Accession Number NP_004426), isoform 1 of protein AIF (NP_004199), AMID (NP_116186), HSP70-1 protein (NP_005336), cyclophilin A (NP_066953), DNA topoisomerase II α (NP_001058). Four internet server tools specialized in predicting cellular localization of proteins from their amino acid sequences were employed: PSORT II [27,28], WoLF PSORT [29], MultiLoc [30], and CELLO v.2.5 [31].

### Fluorescence microscopy

Image data acquisition was done using the high-resolution cytometer (HRCM) developed in our laboratory [25,32,33]. The HRCM system was controlled by FISH 2.0 software or by new Acquiarium 1.0 software, both developed in our laboratory [32-34]. Immunostaining was done using fluorescently labeled primary antibodies labeled using kits with Alexa Fluor dyes 488 and 568 (Invitrogen – Molecular Probes). Rabbit polyclonal anti-human AIF and endoG (Abcam) and mouse monoclonal anti-human cyclophilin A and DNA topoisomerase II α (Tebu-bio) primary antibodies were used. Cells were usually grown in Lab-Tek chambers (Nunc) or grown on coverglass in petri dish. Samples were fixed using 4% paraformaldehyde. Obtained image data were deconvolved using the plugins Diffraction PSF 3D and Iterative Deconvolve 3D for ImageJ software [35].

### Molecular modeling

Prediction servers can use the known 3D protein structure with amino acid sequence homologous to the protein of interest and predict its 3D structure based on this homology. We used servers Phyre [36,37] and M4T [38] for such 3D structure modeling after intensive testing of various servers. Visualization of 3D protein structures was done using software UCSF Chimera [39]. The server tool MolProbity [40] was used for protein model validation and refinement.

### Prediction of interactions

Interaction prediction analysis was conducted using two web server tools for predicting interaction sites in proteins from their 3D structure. Cons-PPISP [41] for prediction of protein-protein interaction residues and DISPLAR [42] for prediction of DNA binding residues.

### Molecular docking

We accomplished the molecular docking using one of the most modern server docking tools PatchDock with refinement tool FireDock [43,44]. For molecular docking were used optimized PDB files of 3D structures from RCSB Protein Data Bank (for AIF it was PDB ID 1m6i, for B-DNA it was PDB ID 1bna, and for cyclophilin A it was PDB ID 1W8M) or our prepared models. The 10 best solutions were saved and submitted to following analysis and refinement by FireDock tool. Again the best 10 solutions and calculations were also saved and then studied.

## Results

### Sequence analysis and prediction of subcellular locations of proteins

**Figure 1:**
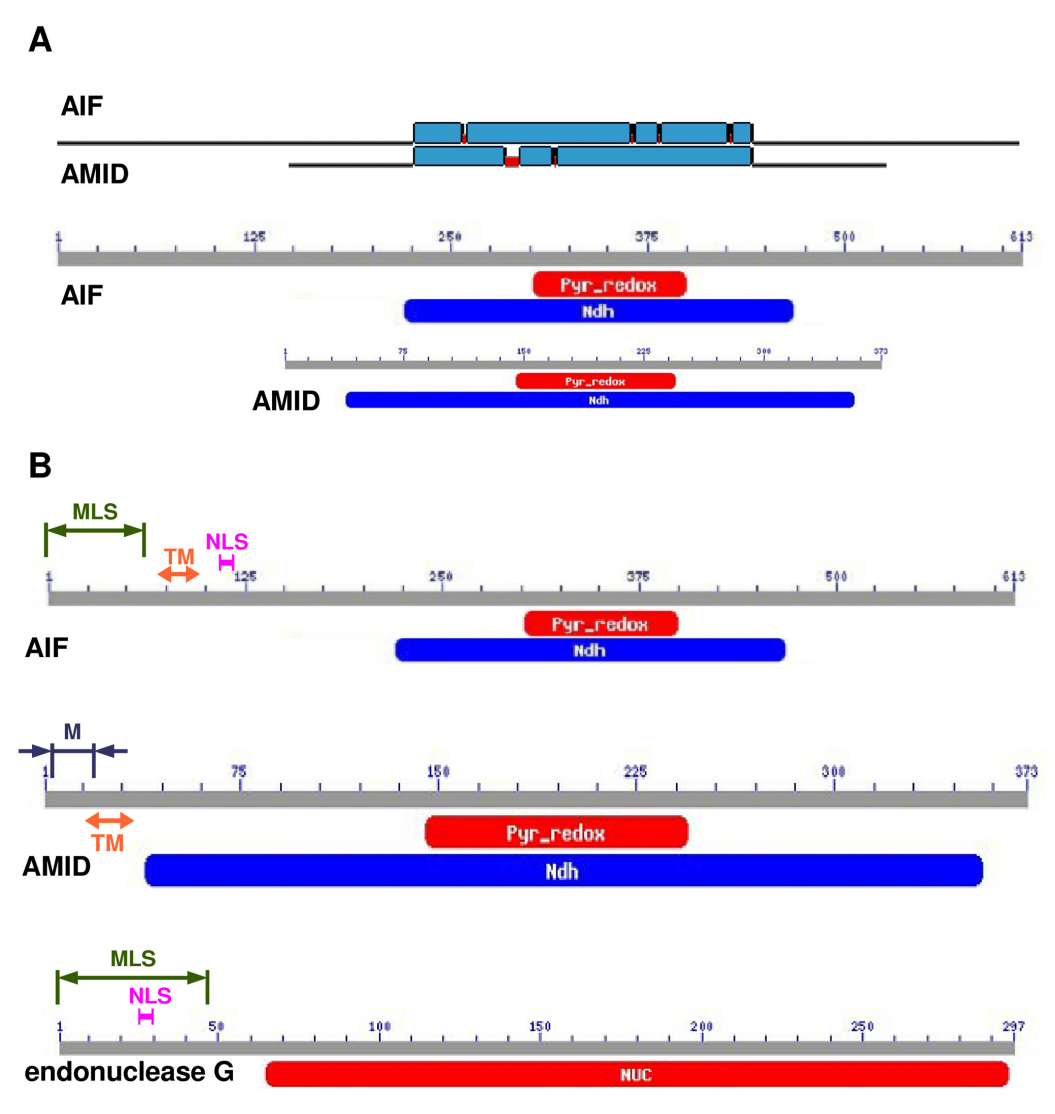
**Sequence analysis and prediction of cellular locations of proteins**. (A) Homology of sequences of AIF and AMID with detected conserved domains. (B) Sequences of AIF, AMID, and endoG with detected conserved domains and predicted sites. Pyr_Redox – NADH binding domain within a larger FAD binding domain of pyridine nucleotide-disulphide oxidoreductase; Ndh – FAD-containing subunit of NADH dehydrogenase; Nuc – DNA/RNA non-specific endonuclease; MLS – mitochondrial localization sequence; NLS – nuclear localization signal; M – N-myristoylation-allowing motif; TM – transmembrane region.

We analyzed the amino acid sequences of AIF and AMID by BLAST server using tool Search Conserved Domains and using tool Blast 2 sequences [45] to study the alignment and the conserved domains of these two proteins (Fig. 1A). Results clearly show that sequence homology of these two proteins is restricted to Ndh (NADH dehydrogenase, FAD-containing subunit) conserved domain. Predictions of the subcellular locations of endoG, AIF, AMID, cyclophilin A, HSP70-1, and DNA topoisomerase II α were made using four prediction servers: PSORT II, WoLF PSORT, MultiLoc, and CELLO. Although most of the analyzed proteins have known cellular location, we nevertheless employed these server tools to validate the accuracy of a final prediction summary based on the combined results of these tools (Table 1). These results clearly indicate that the predicted cellular location of endoG and AIF is in the mitochondrion, cyclophilin A and HSP70-1 in the cytoplasm, and DNA topoisomerase II α in the nucleus. For AMID, the most probable subcellular location was predicted to be the cytoplasm (Table 1). The individual PSORT II algorithms revealed the MLS in endoG sequence to be the N-terminal 48-amino-acid cleavable signal peptide [46,47] and the nuclear localization signal (NLS) at position 24 [48] inside the predicted MLS (Fig. 1B). In the sequence of AIF isoform 1 we predicted the MLS to be the N-terminal 61-amino-acid cleavable signal peptide [46,47], the NLS at positions 106 – 112 [48], and one transmembrane segment between positions 68 – 84 (Fig. 1B) [27]. The PSORT II algorithms discovered an N-myristoylation-allowing motif [49] which would potentially permit incorporation of AMID into various cellular membranes [50] and one transmembrane segment between positions 11 – 33 by TMHMM 2.0 server (Fig. 1B) [51]. N-myristoylation-allowing motif was also detected by two other bioinformatic tools – the Myristoylator [52] and the NMT Predictor [53], which defined the motif to be the amino acid sequence GSQVSVESGALHVVIVG starting at position 2. In the sequence of HSP70-1 were detected NLS at positions 246 – 273 and 594 – 597. PSORT II and TMHMM 2.0 found nothing of interest for cyclophilin A. In the sequence of DNA topoisomerase II α were predicted several NLS between amino acids 632 to 1468.

**Table 1:** Prediction of cellular localization.

Endonuclease G	PSORT II (%)	WoLF PSORT (%)	MultiLoc (%)	Cello (reliability)
mitochondrion	**30.4**	**25.9**	**98.0**	**3.08**
cytoplasm	*34.8*	6.9	0.0	0.33
endoplasmic reticulum	8.7	-	-	0.03
Golgi apparatus	-	10.3	-	0.01
lysosome/vacuole	8.7	13.8	-	0.03
peroxisome	-	15.5	1.0	0.20
plasma membrane	8.7	-	-	0.40
nucleus	8.7	3.4	-	0.43
cystoskeleton	-	-	-	0.01

AIF				

mitochondrion	**39.1**	**69.0**	**97.0**	**2.53**
cytoplasm	26.1	8.6	1.0	0.40
endoplasmic reticulum	17.4	-	-	0.01
Golgi apparatus	13.0	-	-	0.02
lysosome/vacuole	4.3	-	-	0.01
peroxisome	-	6.9	2.0	0.14
plasma membrane	-	6.9	-	0.04
nucleus	-	5.2	-	0.04
cystoskeleton	-	-	-	0.00

AMID				

mitochondrion	8.7	3.1	7.0	*1.58*
cytoplasm	**60.9**	**12.5**	**59.0**	**0.58**
endoplasmic reticulum	-	-	-	0.05
Golgi apparatus	13.0	3.1	-	0.04
lysosome/vacuole	4.3	-	-	0.03
peroxisome	-	*12.5*	31.0	0.25
plasma membrane	-	*18.8*	-	0.37
nucleus	4.3	*12.5*	-	0.17
cystoskeleton	-	-	-	0.01

Cyclophilin A				

mitochondrion	4.3	6.3	1.0	0.33
cytoplasm	**60.9**	**79.7**	**93.0**	**4.18**
endoplasmic reticulum	-	-	-	0.09
Golgi apparatus	-	3.2	-	0.01
lysosome/vacuole	4.3	-	-	0.02
peroxisome	-	-	5.0	0.03
plasma membrane	-	-	-	0.03
nucleus	17.4	2.5	-	0.12
cytoskeleton	4.3	-	-	0.00

HSP70-1				

mitochondrion	-	3.2	2.0	0.47
cytoplasm	**73.9**	**59.4**	**36.0**	**3.08**
endoplasmic reticulum	4.3	-	-	0.22
Golgi apparatus	-	-	-	0.04
lysosome/vacuole	-	-	-	0.01
peroxisome	-	-	*61.0 *	0.10
plasma membrane	-	3.2	-	0.02
nucleus	21.7	21.9	-	0.20
cytoskeleton	-	9.4	-	0.02

DNA topoisomerase II α				

mitochondrion	-	1.6	-	0.10
cytoplasm	17.4	31.3	2.0	0.30
endoplasmic reticulum	-	-	-	0.03
Golgi apparatus	-	-	-	0.01
lysosome/vacuole	-	-	-	0.00
peroxisome	-	-	1.0	0.01
plasma membrane	-	-	-	0.11
nucleus	**78.3**	**67.5**	**96.0**	**4.33**
cytoskeleton	-	-	-	0.02

Sequence analysis of localization of endonuclease G, AIF, AMID, cyclophilin A, HSP70-1, and DNA topoisomerase II α. Bold underlined numbers represent final combined prediction result for corresponding protein. Italic underlined numbers mark the highest probabilities, which are different from the final combined prediction result.

### Experimental cellular locations of proteins

**Figure 2:**
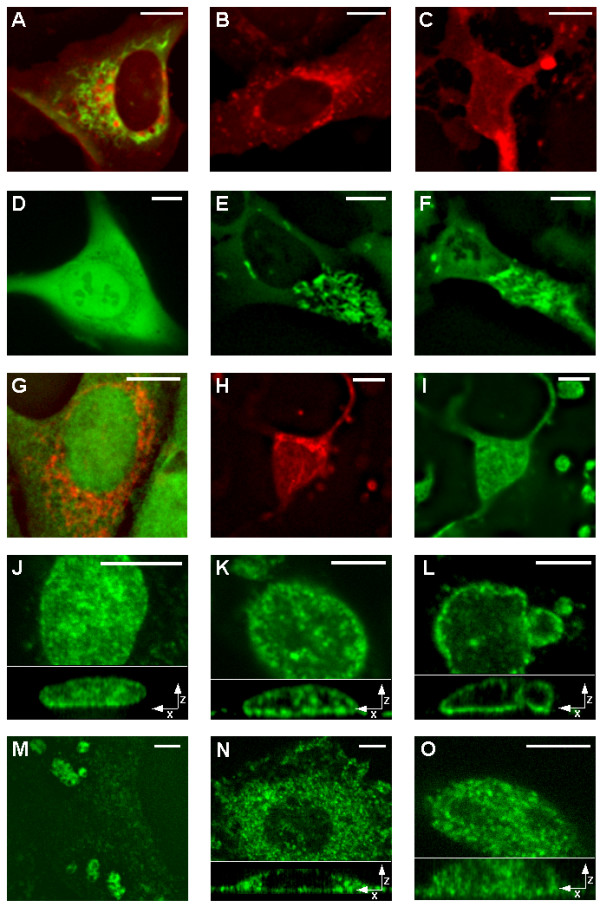
**Living cells expressing fusion proteins and immunostained fixed cells**. Representative fluorescent images of living U-2 OS cells expressing endoG-EYFP (green) and AMID-tHcRed (red) (A), AMID-tHcRed (B), and AMID-tHcRed 6 hours after induction of apoptosis by addition of 200 nM staurosporine (C). Representative fluorescent images of stably transfected living cell U-2 OS over-expressing endoG-EYFP (D), living cell U-2 OS expressing endoG-EYFP before (E) and 6 hours after (F) apoptotic induction by 200 nM staurosporine. Fixed U-OS cells immunostained with fluorescently labeled primary antibodies against human AIF (red) and cyclophilin A (green) (G), U-2 OS cell 6 hours after induction of apoptosis by 200 nM staurosporine immunostained against human AIF (H) and cyclophilin A (I). Representative fluorescent images of fixed U-2 OS cells immunostained with fluorescently labeled primary antibodies against human DNA topoisomerase II α at non-apoptotic condition (J), 3 (K) and 6 (L) hours after induction of apoptosis by 200 nM staurosporine. Representative fluorescent images of fixed U-2 OS cells immunostained with fluorescently labeled primary antibodies against human HSP70-1 after heat shock for 2 hours at 42°C (M), fixed U-2OS cells immunostained against human HSP70-1 before (N) and 6 hours after (O) apoptotic induction by 200 nM staurosporine. Bottom parts of figures J, K, L, N, O contain representative sections of XZ projections of acquired 3D images. Scale bars = 10 μm.

Experimental determination of the cellular locations of studied proteins was conducted either by transfecting living cells with mammalian expression vectors encoding the fusion proteins or by immunostaining of fixed cells by fluorescently labeled primary antibody. Figure 2A shows a typical signal distributions of endoG-EYFP and AMID-tHcRed fluorescence in transfected human living U-2 OS cells. Apparently, AMID and endoG do not colocalize significantly. EndoG is present in mitochondria and AMID is present throughout the cytoplasm apparently on various structures [25]. Figures 2B and 2C show signal distribution of AMID-tHcRed fluorescence in living U-2 OS cell before and 6 hours after induction of apoptosis by 200 nM staurosporine. Figure 2C clearly shows that AMID does not translocate to nucleus. Figure 2D shows the fluorescence signal of endoG-EYFP in one living cell over-expressing endoG which distributed inside nucleus, although the cell is viable and non-apoptotic. Translocation of endoG into the cell nucleus during staurosporine-induced apoptosis is shown in Figures 2E and 2F. We co-immunostained AIF and cyclophlilin A in fixed U-2 OS cells (Fig. 2G). AIF is located to mitochondria and cyclophilin A to the cytoplasm and also to the cell nucleus. Apoptosis induced in these cells resulted into nuclear translocation of AIF (Fig. 2H) and cyclophilin A remained in nucleus and cytoplasm (Fig. 2I). Immunostaining by fluorescent antibodies against DNA topoisomerase II α revealed the expected nuclear localization of the protein (Fig. 2J). Interestingly, after apoptosis induction by 200 nM staurosporine, DNA topoisomerase II α in nucleus displayed distribution similar to chromatin condensation attributed to effect of AIF during apoptosis (Fig. 2K and 2L). HSP70-1 immunostaining showed not only cytoplasmic localization of the protein (Fig. 2N) but also strong nucleoli localization of HSP70-1 after heat shock at 42°C for 2 hours (Fig. 2M). Interestingly, after apoptosis induction by 200 nM staurosporine, HSP70-1 relocated to the cell nucleus (Fig. 2O).

### Molecular modeling

**Figure 3:**
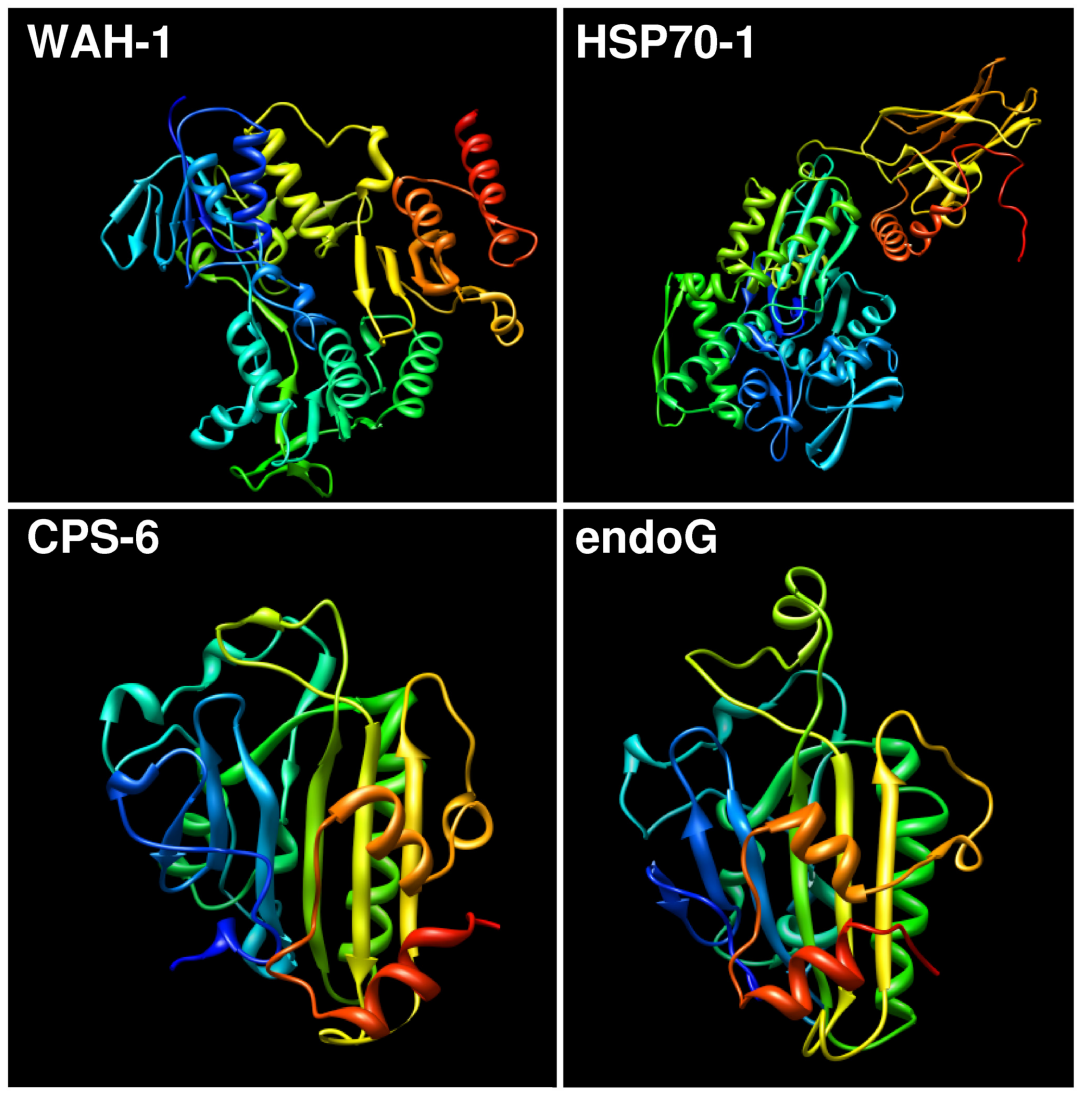
**3D structure models of proteins**. Models for proteins WAH-1 (modeled amino acids 238–700 of 700), CPS-6 (modeled amino acids 57–305 of 308), and endoG (modeled amino acids 65–296 of 297) were calculated using Phyre server. Model of HSP70-1 (modeled amino acids 1–554 of 641) was calculated by server M4T. Structures were visualized using UCSF Chimera software.

We prepared 3D models of protein structure of several proteins using various modeling servers and we selected only the best models ranked by MolProbity [40] server. MolProbity server also refined the structure of models and these refined models were used in our following predictions and molecular dockings. Best endoG model (prepared from 1G8T structure) was prepared by Phyre server [36] (MolProbity score 2.96) and consists of amino acids 65–296 from 297 amino acids (Fig. 3). We also prepared model of HSP70-1 protein (MolProbity score 2.68) by M4T server [38] for amino acids range of 1–554 from 641 amino acids (Fig. 3). Another two models were prepared again by Phyre server for proteins WAH-1 (amino acids 238–700 of 700; MolProbity score 3.02) and CPS-6 (amino acids 57–305 of 308; MolProbity score 3.06) (Fig. 3).

### Prediction of interactions

Using servers cons-PPISP [41] and DISPLAR [42] we predicted the possible binding sites for proteins (cons-PPISP) and DNA (DISPLAR) in the sequences of AIF, endonuclease G, and cyclophilin A. These predictions are based on 3D structures of these proteins. Using cons-PPISP server we found several residues that can form binding sites in the AIF amino acid sequence. The binding residues are in the interval of amino acids 264–498 and at the C-terminus (amino acids 598–608). For endoG we predicted two possible binding regions for amino acids 75–96 and 265–282 (C-terminus). We found a high concentration of possible binding residues at amino acids 88–97 and 116–138 for cyclophilin A. Using DISPLAR server, we were able to detect possible locations for DNA binding residues in the sequence of AIF especially at interval of amino acids 237–254. EndoG sequence showed three intervals containing predicted residues at positions 100–115, 139–153, and 177–188. For cyclophilin A, we detected cluster of predicted residues for DNA binding in the interval of amino acids 54–72.

### Molecular docking

**Figure 4:**
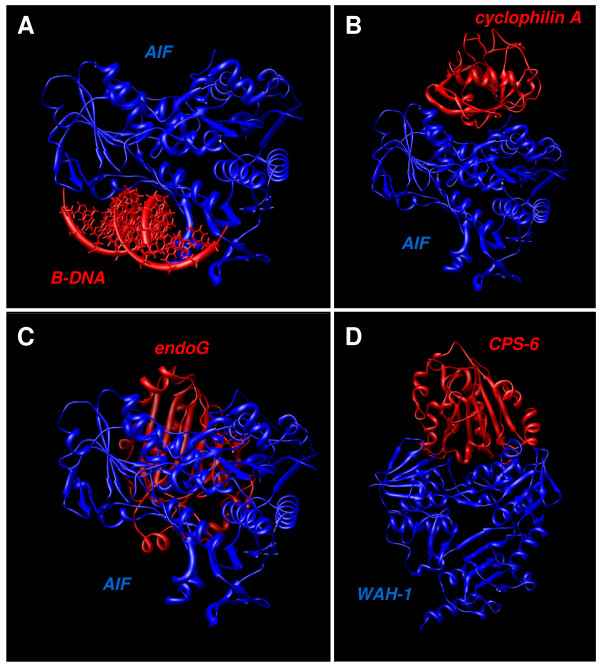
**Molecular docking**. Resulting complexes obtained by molecular docking conducted by PatchDock and refined by FireDock. Figures represent probable binding configuration of AIF with ideal B-DNA (A), AIF with cyclophilin A (B), AIF with endoG (C), and WAH-1 with CPS-6 (D). Structures were visualized using UCSF Chimera software.

**Figure 5:**
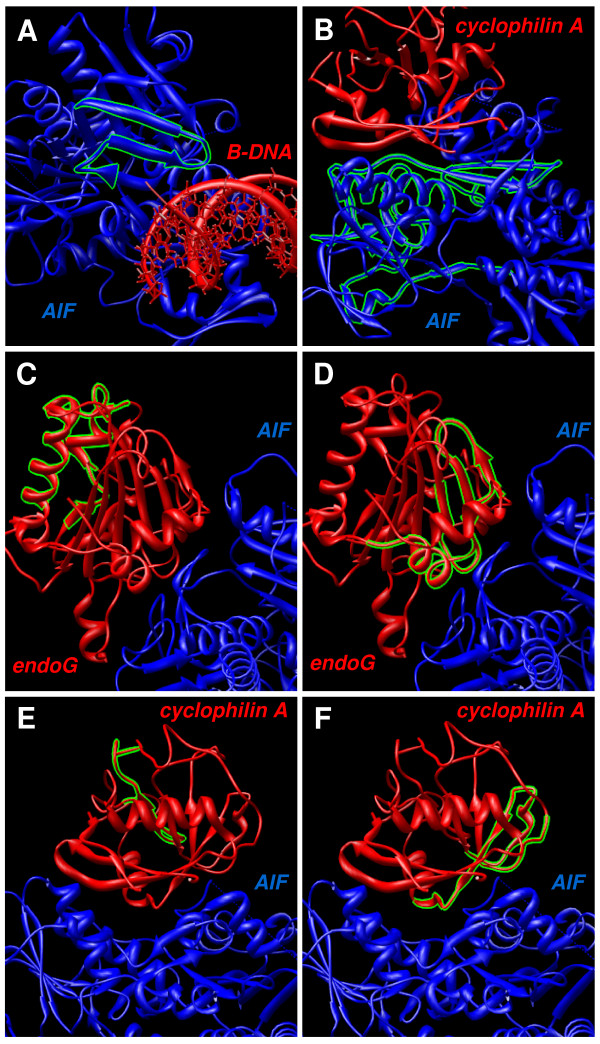
**Predicted binding sites visualization in molecular docking results**. Visualization of predicted binding residues suggested by DISPLAR server for protein-DNA binding and by cons-PPISP server for protein-protein binding. Figures A, C, and E show predicted protein-DNA binding residues in structure of AIF (A), endoG (C), and cyclophilin A (E). Figures B, D, and F show predicted protein-protein binding sites in structure of AIF (B), endoG (D), and cyclophilin A (F). Structures were visualized using UCSF Chimera software.

Docking represents the mathematical calculation of the most probable spatial orientation of two interacting molecules, usually a protein and a small ligand, two interacting proteins, or DNA and protein. Various parameters are calculated to evaluate possibility of such interaction. For molecular docking we used new server PatchDock [43] with refinement tool FireDock [44]. We modeled the proposed interaction of AIF with B-DNA [18] using these tools and best docking model is shown (Fig. 4A). Global energy function of this complex was calculated by FireDock server to be -16.84 of relative units (this value is considered to be related to free binding energy and higher negative value means higher free binding energy and thus higher interaction probability). Another experimentally shown interaction pair we studied was AIF and cyclophilin A (Fig. 4B) [19]. Global energy value of this complex was -8.41. Next we modeled the possible interaction of AIF and endoG (Fig. 4C) resulting in global energy value -26.27 for this complex. Lastly, we modeled experimentally proved interaction of analogues of AIF and endoG, WAH-1 and CPS-6 (Fig. 4D) [17]. Resulting global energy was -13.11. Figure 5 shows parts of modeled docking complexes described above with focus on visualization of binding sites. In green color are shown residues predicted by cons-PPISP or DISPLAR servers mentioned above that can form binding sites for protein-protein and protein-DNA interactions.

## Discussion

We clearly show that sequence homology of AIF and AMID is restricted to Ndh conserved domain, that corresponds to oxidoreductase function and not to apoptotic function of AIF, which was shown to reside in large C-terminal part of AIF sequence, which is totally missing in AMID sequence (Fig. 1A) [54]. AMID sequence contains, aside from Ndh conserved domain and N-terminal part, only very small C-terminal part consisting of only several residues and thus it is highly improbable that AMID will act similarly as AIF during apoptosis. We correctly predicted that endoG and AIF would localize to the mitochondria, HSP70-1 and cyclophilin A to the cytoplasm, and DNA topoisomerase II α to the nucleus (Table 1). The cytoplasm was found as the most probable cellular location for AMID (Table 1). This result is in agreement with previous observations [25], although some of them described the distribution of AMID in the cytoplasm differently [5,24]. No recognizable localization signal was found in the AMID amino acid sequence. However, the MLS was detected as the first 48 amino acids of the endoG sequence and as the first 61 amino acids of the AIF sequence [55]. This finding corroborates the many published observations that these proteins are translocated into the nucleus during apoptosis [7,15,56]. Interestingly, the predicted NLS of endoG is located within the MLS, so that once endoG enters the mitochondrion the NLS will be cleaved together with the MLS. NLS was found in sequences of DNA topoisomerase II α, which was expected, due to function of this protein. Interestingly, NLS was also found in the sequence of HSP70-1 suggesting its possible role in nucleus. HSP70-1 was found in nucleoli after the heat shock [57]. The algorithms and TMHMM 2.0 server also predicted that the sequence of endoG, cyclophilin A, HSP70-1 and DNA topoisomerase II α do not contain any transmembrane regions. However, PSORTII algorithms and TMHMM 2.0 revealed that AIF and AMID sequences contain one predicted transmembrane region thus suggesting that AIF and AMID could be membrane proteins [11,58]. When apoptosis is induced the AIF protein is spliced probably by calpain I and looses its N-terminal part that contains the predicted transmembrane region and AIF is thus released into the intermembrane space and out of mitochondria [59]. Sequence of cyclophilin A showed no recognizable regions and is therefore interesting, that this protein is often found in nucleus, which may support the possibility that cyclophilin A interacts with AIF which contains NLS and together they translocates to nucleus [19]. The lipid anchoring is also possible for AMID, mediated by its identified putative N-myristoylation site. This site allows AMID to incorporate into various cellular membranes. Our findings thus predict that AMID is by N-terminal part incorporated into cellular membranes from cytoplasmic side.

Fluorescence imaging of living cells and microscopy of immunostained fixed cells (Fig. 2) confirmed the predicted localization of studied proteins (Table 1). AIF and endoG localized to mitochondria (Fig. 2A, E, G) and DNA topoisomerase II α to nucleus (Fig. 2J). DNA topoisomerase II α was also found to relocate during apoptosis similarly to chromatin condensation organized by AIF (Fig. 2K and 2L). This can point to possible interaction of DNA topoisomerase II α with AIF or it simply binds to condensed chromatin during apoptosis. Apart from cytoplasmic distribution, cyclophilin A surprisingly showed strong nuclear staining (Fig. 2G) maintained even during apoptosis (Fig. 2I), although its sequence does not contain any recognizable signals or motifs. Cyclophilin A can probably bind other protein that can translocate to nucleus even at non-apoptotic conditions. Immunostaining of HSP70-1 after strong heat shock showed significant nuclear localization of HSP70-1 into nucleoli (Fig. 2M) as was already observed [57]. Localization of HSP70-1 in non-apoptotic conditions was found to be cytoplasmic as predicted (Fig. 2N), but suprisingly during apoptosis HSP70-1 translocated into the cell nucleus (Fig. 2O), which even more strongly supports the possible role of this protein in apoptosis and it also corresponds to our prediction results. Cells of normal morphology expressing high levels of endoG-EYFP were identified in stably transfected clones. The signal comprised not only the mitochondrial fluorescence but also a strong confluent fluorescence in the cytoplasm and even in the nuclear chromatin (Fig. 2D). Such cells showed no morphological apoptotic changes. Thus the presence of endoG in the cytoplasm and nucleus was not sufficient to induce apoptotic chromatin degradation in the cells. This confirms our bioinformatic predictions about endoG, that the NLS of endoG can transport endoG-EYFP into the nucleus when the protein is highly overexpressed in the cytoplasm. These findings present the experimental evidence that the mere presence of endoG in the nucleus is not sufficient to initiate DNA cleavage and, although it is a nuclease, endoG apparently needs to be activated to degrade DNA in living cells [6,60]. The fluorescence signal of AMID-tHcRed was distributed throughout the cytoplasm, although not diffuse as was suggested [24]. AMID was found to localize to unidentified regions throughout the entire cell (Fig. 2A and 2B). AMID-tHcRed localized close to the nuclear membrane, possibly with the Golgi apparatus and/or the endoplasmic reticulum; it was also associated with small vesicles in the cytoplasm and possibly with the plasma membrane and it does not translocate to the nucleus during apoptosis (Fig. 2C) [25,26]. Cells overexpressing AMID-tHcRed were viable with normal morphology, and overexpression of AMID was not sufficient to induce apoptosis; thus, our results challenge a previous report that overexpression of AMID could induce apoptotic degradation of chromatin and the loss of structurally differentiated mitochondria [5].

We prepared 3D structural models for proteins without known 3D structure (Fig. 3). Both, these refined models and known protein structures, were used for *in silico *prediction of locations of binding residues for DNA and proteins. We studied especially molecules that were shown to interact with AIF or are possible candidates for such interaction. Especially we studied the interaction of AIF with B-DNA (Fig. 4A) and cyclophilin A (Fig. 4B) using molecular modeling. We also studied the hypothetical possible interaction of AIF with endoG (Fig. 4C), based on know interaction of their analogues WAH-1 and CPS-6 from *Caenorhabditis elegans *(Fig. 4D) [17]. The resulting values produced by FireDock server tool are suggesting that interaction of AIF with endoG is very probable due to the high negative value of global energy function, comparable to other known interactions. We visualized the predicted binding residues from servers cons-PPISP and DISPLAR in our molecular docking complexes (Fig. 5). This novel visualization clearly shows, that molecular docking results and binding residues predictions corresponds very well, thus supporting results of each other and verifying the binding residues locations and molecular binding spatial configuration of the studied proteins.

Our results show great possibilities of in silico methods and image analysis to understand the localization, interactions and functions of proteins. Our results present new data about the structure, localization, functions and interactions of proteins AMID, AIF, endonuclease G, and other apoptosis-related proteins.

## Competing interests

The authors declare that they have no competing interests.

## Authors' contributions

MV carried out all bioinformatic studies, live-cell fluorescence microscopy, and drafted the manuscript. MZ carried out fluorescence microscopy of fixed samples. JA carried out design of some experiments and preparation of samples. VU, PM, and MK constructed the automated imaging platform for fluorescence microscopy and developed the imaging and analysis software controlling this platform.
